# Cyclooxygenase 2-Regulated Genes an Alternative Avenue to the Development of New Therapeutic Drugs for Colorectal Cancer

**DOI:** 10.3389/fphar.2020.00533

**Published:** 2020-04-29

**Authors:** Alicia M. Hidalgo-Estévez, Konstantinos Stamatakis, Marta Jiménez-Martínez, Ricardo López-Pérez, Manuel Fresno

**Affiliations:** ^1^Faculty of Biomedical and Health Sciences, Universidad Europea de Madrid, Madrid, Spain; ^2^Centro de Biología Molecular Severo Ochoa, Consejo Superior de Investigaciones Científicas, Universidad Autónoma de Madrid, Madrid, Spain; ^3^Instituto Sanitario de Investigación Princesa, Madrid, Spain

**Keywords:** cyclooxygenase, prostaglandin, Colon cancer, therapy, tumor development, metastasis, effector genes/proteins

## Abstract

Colorectal cancer (CRC) is one of the most common and recurrent types of cancer, with high mortality rates. Several clinical trials and meta-analyses have determined that the use of pharmacological inhibitors of cyclooxygenase 2 (COX-2), the enzyme that catalyses the rate-limiting step in the synthesis of prostaglandins (PG) from arachidonic acid, can reduce the incidence of CRC as well as the risk of recurrence of this disease, when used together with commonly used chemotherapeutic agents. These observations suggest that inhibition of COX-2 may be useful in the treatment of CRC, although the current drugs targeting COX-2 are not widely used since they increase the risk of health complications. To overcome this difficulty, a possibility is to identify genes regulated by COX-2 activity that could give an advantage to the cells to form tumors and/or metastasize. The modulation of those genes as effectors of COX-2 may cancel the beneficial effects of COX-2 in tumor transformation and metastasis. A review of the available databases and literature and our own data have identified some interesting molecules induced by prostaglandins or COX-2 that have been also described to play a role in colon cancer, being thus potential pharmacological targets in colon cancer. Among those mPGES-1, DUSP4, and 10, Programmed cell death 4, Trop2, and many from the TGFβ and p53 pathways have been identified as genes upregulated in response to COX-2 overexpression or PGs in colon carcinoma lines and overexpressed in colon tumor tissue. Here, we review the available evidence of the potential roles of those molecules in colon cancer in the context of PG/COX signaling pathways that could be critical mediators of some of the tumor growth and metastasis advantage induced by COX-2. At the end, this may allow defining new therapeutic targets/drugs against CRC that could act specifically against tumor cells and would be effective in the prevention and treatment of CRC, lacking the unwanted side effects of COX-2 pharmacological inhibitors, providing alternative approaches in colon cancer.

## COX-2 in Colorectal Cancer

Colorectal cancer (CRC) is the third most common type of cancer and the second leading cause of cancer-related death in the world, with around 900,000 deaths per year (Globocan, 2018; Keum and Giovannucci, 2019). Even in patients who have undergone tumor resection, 40–50% relapse and die of metastases, being the overall 5-year survival less than 60%. Despite this health burden, only few therapeutic options exist for this disease, including combination of chemotherapy and anti-angiogenic agents (Kuipers et al., 2015; Karpisheh et al., 2019). Thus, currently available therapeutic options do not achieve the desired efficiency and development of new strategies is required.

There are two types of CRC: hereditary or familial colon cancer and sporadic colon cancer (Sancho et al., 2004; Ma et al., 2018). Nonetheless, sporadic colon cancer is the most common with an incidence of 75–80% in diagnosed cases. The alterations generated in two main molecular pathways subdivide this type of cancer. In 80–85% of sporadic CRCs there are mutations or deletions in suppressor genes such as KRAS, APC, DCC, and p53, promoting chromosomal instability (Sancho et al., 2004; Moran et al., 2010). In 1990, Fearon and Vogelstein proposed that the mutation in three or four of the aforementioned oncogenes and/or suppressor genes is necessary to initiate the tumorigenesis process, where the final properties of the tumor are determined from the accumulation and order in which changes appear (Fearon and Vogelstein, 1990). Later, it was described how the epithelial cells of the CRC acquire the genetic alterations in a strict order and involve the Wnt and TGFβ signaling pathways in cancer promotion (Arends, 2000). On the other hand, the non-canonical route, also called “mutator”, comprises 15–20% of sporadic CRCs and depends mainly on the alteration due to the instability of microsatellite sequences (MSI, Microsatellite Sequences Instability) in the genome due to the accumulation of mutations in the genes for rupture repair (MMR, Mismatch Repair) produced during DNA replication (Imai and Yamamoto, 2008).

Many clinical trials and epidemiological studies have suggested that the use of non-steroidal anti-inflammatory drugs (NSAIDs), which are classic inhibitors of COX enzymatic activity reduce the risk of developing cancer in general, and more specifically in CRC (reviewed in (Thun et al., 2012; Rothwell, 2013; Piazuelo and Lanas, 2015; Patrignani and Patrono, 2016). Taking together all these data strongly suggest that Cox targeting is a mechanism for cancer prevention.

COX enzymes catalyse the rate limiting step in the conversion of arachidonic acid, derived from membrane phospholipids by phospholipase A2, into prostanoids (Williams and Dubois, 1996). Two main isoforms of this enzyme are known: COX-1 encoded by prostaglandin-endoperoxide synthase 1 (*PTGS1*) and COX-2 (encoded by *PTGS2*). Although both proteins show the same cyclooxygenase and peroxidase activity, their differences are found in substrates, cell expression, inhibition, and intracellular localization and induction (Garavito et al., 2002). COX-1 is constitutively expressed in most tissues and involved in physiological processes. However, COX-2 is not usually detectable in normal tissues but is induced by numerous cytokines, growth factors, hormones, and tumor promoters. Prostanoids modulate immune responses, renal function, blood clotting and play a role in many pathological conditions, such as inflammation, pain, fever, swelling, etc. (Iniguez et al., 2003; Iniguez et al., 2008; Sreeramkumar et al., 2012).

Considerable amounts of evidence in clinical settings further support a role of COX-2 in colorectal carcinogenesis and tumor progression [reviewed in (Subbaramaiah and Dannenberg, 2003; Wang and Dubois, 2010)]. Thus, COX-2 is expressed early during the adenoma-carcinoma sequence that occurs in CRC, suggesting an important role of this enzyme in colorectal carcinogenesis. COX-2 expression is upregulated in human colorectal adenocarcinomas when compared with normal adjacent colonic tissue. Moreover, polymorphisms of the *PTGS2* gene were associated with risk of CRC (Cox et al., 2004; Agundez et al., 2015). The use of Min/+ mice as a CRC model showed elevated levels of COX-1 and COX-2 in sporadically formed adenomas (Williams et al., 1996) and inhibition of COX-2 resulted in a substantial decrease in intestinal polyp number and size (Jacoby et al., 2000). In the same way, Apc716 mice developed a smaller number and size of tumor polyps when the COX-2 gene was eliminated (Oshima et al., 1996).

In addition, many reports of colorectal tumor cells either overexpressing COX-2 or having it silenced have correlated increased COX-2 expression with their invasive and metastatic properties in xenografted tumors in mice (Tsujii and Dubois, 1995; Tsujii et al., 1997; O’mahony et al., 1999; Chen et al., 2001; Sun et al., 2002; Yoshimoto et al., 2002; Charames and Bapat, 2006; Strillacci et al., 2006; Stamatakis et al., 2015). However, the molecular mechanisms by which COX-2 expression in intestinal epithelial cells leads to that phenotype have not been fully elucidated yet.

In view of the abundant biological and phenotypic evidence, several clinical trials have been performed, aimed to evaluate the efficacy of specific inhibitors of COX-2 (COXIBs) (Fitzgerald and Patrono, 2001) to prevent or delay the onset (or recurrence) of tumors in high-risk patients, including those with prior removal of colon tumors. These studies indicate that specific inhibition of COX-2 prevents the (re)appearance of tumors but also show cardiovascular side-effects [reviewed in (Sinicrope, 2006; Bertagnolli, 2007)].

Recent studies, remark the role of COX-2 in constitutive IDO1 expression by human tumors and substantiate the use of COX-2 inhibitors to improve the efficacy of cancer immunotherapy, either by reducing constitutive IDO1 expression, which contributed to the lack of T-cell infiltration in tumors that fail to respond to immunotherapy (Hennequart et al., 2017), or by synergizing with anti-checkpoint antibodies (Zelenay et al., 2015).

For all of the above, the study of the expression of COX-2 in the different phases of tumor progression and metastasis and the finding of new signaling pathways triggered by this enzyme are essential in order to develop new drugs that inhibit the effects of COX-2 both in cancer prevention and therapy (Rizzo, 2011).

## Prostanoids in Colon Cancer

The activity of cyclooxygenases (COX) is coupled to several terminal synthases that produce the five primary different prostanoids: prostaglandin D2 (PGD_2_), prostaglandin E2 (PGE_2_), prostaglandin F2α (PGF_2α_), prostaglandin I2/prostacyclin (PGI_2_), and thromboxane A2 (TXA_2_) (Iniguez et al., 2003; Iniguez et al., 2008) being also some of them implicated in colon cancer.

### PGE_2_ in Colon Cancer

Among the prostanoids, PGE_2_ has been proposed as the principal prostanoid promoting tumor growth and survival in CRC. PGE_2_ is present in the healthy colon but its levels are elevated in CRC (Pugh and Thomas, 1994; Cathcart et al., 2012; Zuo and Shureiqi, 2013) and correlate with tumor size (Pugh and Thomas, 1994; Yang et al., 1998) and disease progression (Karpisheh et al., 2019). The elevation of PGE_2_ levels may favor tumor growth and invasion *via* several mechanisms as stimulating cell proliferation, inducing local immunosuppression, inhibiting apoptosis, promoting angiogenesis, and increasing cell migration and invasion as well as drug resistance in colon cancer cells (Iniguez et al., 2003; Corral et al., 2007; Greenhough et al., 2009; Wang and Dubois, 2010; Karpisheh et al., 2019). Moreover, there is a positive feedback loop between COX-2 and PGE_2_, in which COX-2 induces PGE_2_ production, and that in turn increases further the expression of COX-2 in colon cancer cells (Stamatakis et al., 2015; Karpisheh et al., 2019).

Three PGE_2_ synthases from PGH_2_ have been described (Hara et al., 2010), two microsomal, mPGES1, mPGES-2, and the cytoplasmic cPGES, encoded by the PTGES, PTGES2, and PTGES3 genes, respectively. MPGES1 expression has been associated to CRC incidence and prognosis (Seo et al., 2009; Sasaki et al., 2012) and has been proposed to cooperate with COX-2 to enhance tumor growth (Kamei et al., 2003). cPGES is a non-inducible isoform, constitutively expressed in most tissues and associated with COX-1 activity while maintaining the production of PGE_2_ (Tanioka et al., 2000).

Notably, we found co-localization of COX-2 and MPGES1 in human CRC biopsies and that PTGS2 and PTGES gene expression levels strongly correlate in human microarray databases. Interestingly, we have described the joint induction of mPGES-1 and COX-2 by PGE_2_ due to the involvement of the transcription factor EGR1 (Early Growth Response Protein 1), representing a positive feedback loop between COX-2, mPGES-1, and PGE_2_ (Stamatakis et al., 2015). Moreover, mPGES-1 overexpressing carcinoma cell lines have increased tumorigenic capacity *in vivo* indicating that high levels of any of the two enzymes is sufficient to enhance CRC growth (Stamatakis et al., 2015). Our results demonstrate that mPGES1 is induced by COX-2 overexpression, *via* autocrine PGs release, likely PGF_2α_ through an EGR1-dependent mechanism in colon carcinoma.

Thus, mPGES1 has gained attention recently as alternative target to COX-2 for CRC chemoprevention and chemotherapy (Sasaki et al., 2015). In this sense, mPGES1 inhibitors have demonstrated promising effects in inhibiting colon carcinoma growth (Dixon et al., 2013; Wang et al., 2018; Karpisheh et al., 2019), that may circumvent the *in vivo* cardiovascular toxicity associated with COX-2 inhibitors.

PGE_2_ acts through its binding to prostanoid-E (EP) receptors. Four subtypes of EP receptors (EP1, EP2, EP3, EP4) have been identified. Their tissue-specific expression and activation trigger different signaling pathways that favor, for example, the cellular proliferation of the intestinal epithelium (Takafuji et al., 2000). EPs belong to the family of G-protein coupled receptors (GPCRs) and transduce PGE_2_ signaling through different intracellular messengers. EP2 and EP4 preferably increase intracellular levels of cyclic adenosine monophosphate (cAMP) while signaling by EP3 mostly mobilizes cAMP and the activation of EP1 mobilizes calcium (Ca^2+^) (Regan, 2003; Alfranca et al., 2006). In different mice models of colon cancer, genetic deletion of the EP1 (Kawamori et al., 2005), EP2 (Ma et al., 2015), and EP4 receptors inhibits colonic tumorigenesis (Mutoh et al., 2002).

### Prostaglandin 2α in Colorectal Cancer

PGF_2α_ is mainly produced by the activity of PGF synthase (PGFS) from PGH_2_ and specifically activates the GPCR type FP receptor (Breyer et al., 2001; Komoto et al., 2006). Signaling through the two FP isoforms leads to the activation of phospholipase C (PLC) and, consequently, an increase in intracellular Ca^2+^ and the activation of protein kinase C (PKC). However, PGF_2α_ can be synthesized from other PGs such as PGE_2_ and PGD_2_, and can signal through other receptors such as EP1 and EP3, thus varying the intracellular signaling pathways (Breyer et al., 2001; Komoto et al., 2006). For example, induction of COX-2 through FP can occur through Rho activation and transcription mediated by β-catenin/Tcf (Fujino and Regan, 2003). Along those lines, elevated levels of cAMP have been detected in cells isolated from colonic crypts after treatment with PGF_2α_ (Collins et al., 2009).

There is very little evidence of the possible role of PGF_2α_ in CRC other than its higher levels, together with PGE_2_ (Nugent et al., 1996). Also, in the tumor tissue of patients with CRC, a differential regulation of the cancer-dependent FP receptor has been detected (Gustafsson et al., 2007). PGF_2α_ also promotes the invasion and mobility of epithelial cells of adenomas and colon carcinomas in collaboration with PGE_2_ (Qualtrough et al., 2007).

Recently, we have shown that colorectal tumor cells produce PGF_2α_ though COX-2 and have provided some evidence that PGF_2α_ may also play a role in colorectal tumorigenesis (Stamatakis et al., 2015). Colorectal adenoma and carcinoma-derived cell lines secrete PGF_2α_ while they also express FP indicating potential autocrine effects. Interestingly, this secreted PGF_2α_ was able to increase mPGES1 but also COX-2 in colon carcinoma cells, *via* FP receptor and EGR1, further enhancing PGE_2_ levels indicating a positive feedback loop between COX-2/mPGES1/PGE_2_ and PGF_2α_ (Stamatakis et al., 2015). However, PGF_2α_ failed to directly induce cell proliferation in CRC cell lines (Cassano et al., 2000).

### Other Prostanoids

The roles of the other COX-derived prostanoids in CRC tumors, as well as in carcinoma cell lines remains poorly understood (Cathcart et al., 2011). It has been reported that the levels of some prostaglandin receptors, such as IP (PGI_2_ receptor) and DP (PGD_2_ receptor), were reduced in CRC cells and the downregulation of one of them, DP2, has been related to differentiation of healthy epithelium to tumor (Gustafsson et al., 2007). The expression of thromboxane synthase (TXS), the enzyme that catalyses the conversion of PGH2 to TXA_2_, was significantly increased in CRC tumors compared to normal tissue. Furthermore, genetic or pharmacological reduction of TXS diminishes proliferation in CRC cell lines (Ekambaram et al., 2011). In addition it has also been described that the lack of the enzyme 15-PGDH, responsible for the inactivation of prostaglandins and lipoxins, is associated with CRC (Backlund et al., 2005) and together with the increase in the PGE_2_ synthesis, induces tumor formation in Min (multiple intestinal neoplasia) CRC model in mice. (Myung et al., 2006). Besides the loss of 15‐PGDH and induction of this enzyme has been associated with the suppression of inflammation-driven colon carcinogenesis in mice (Choi et al., 2014).

## Prostaglandin Pathway and Colorectal Cancer

**Figure 1:**
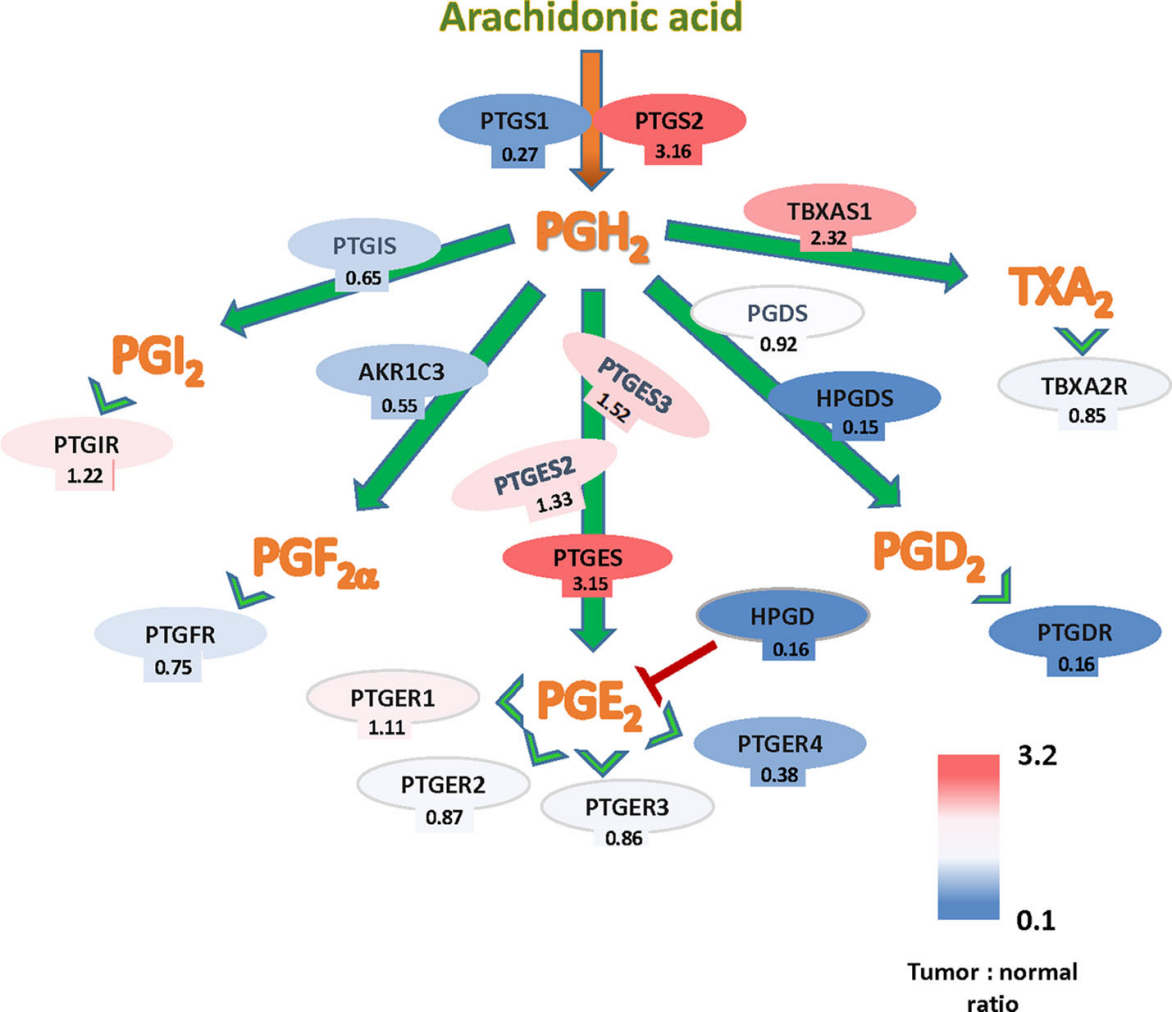
Arachidonate-prostanoids pathway gene expression in colorectal cancer (CRC) tumors. Shown are mRNA expressions in tumors of enzymes and receptors from the arachidonate-prostanoids pathway. Numbers indicate the fold differences with normal adjacent tissue based on the The Cancer Genome Atlas (TCGA) Colon Cancer dataset.

The potential implication of the Cox-PG synthases-PG receptors in CRC can be also studied through expression levels of the pathway´s components using The Cancer Genome Atlas (TCGA) gene expression data. A summary of these findings is shown in **Figure 1**, based on the TCGA Colon Cancer dataset. These analyses, comparing tumor and normal colon epithelia, showed that gene expressions from several members of this pathway are upregulated in tumors, namely *PTGS2*, *PTGES*, and *TBXAS1*, indicating that the biosynthetic and signaling pathways of PGE_2_ and TXA_2_ are favored in established tumors. Conversely, expression downregulation in tumor tissue was observed for genes encoding components of the pathway leading to the signaling of other prostanoids, but most notably of *HPGD* that encodes the PGE_2_-inactivating enzyme 15-PGDH. Analysis with other different datasets, i.e., the OncoMine microarray database (Compendia Bioscience), resulted in very similar data regarding *PTGS2*, *PTGES* (Stamatakis et al., 2015). Together, those analyses confirm the important role of COX2-2 and PGE_2_ in colon cancer. Nonetheless, since COX is the rate-limiting enzyme in the synthesis of all prostanoids, an increased expresion of this enzyme could result in the elevation of other prostanoids besides PGE_2_.

## COX-2 Downstream Genes in CRC

As mentioned above, the treatment of patients with NSAIDs or COXIBs greatly decreases tumor progression in a number of clinical trials in CRC. However, since general inhibitors of COXs, such as aspirin, can cause gastrointestinal bleeding problems and specific inhibitors of COX-2 increase the risk of cardiovascular disease, alternative treatments are needed targeting the downstream effector of COX-2 protumoral activity, likely minimizing side effects, and if possible aiming exclusively to cancer cells (Cathcart et al., 2011).

Although PGE_2_ blockade may represent a good therapeutic option, other PGs besides PGE_2_ could be responsible for COX-2 effect in colon carcinoma cells. Besides, although prostanoids may modulate the growth and invasiveness of colon carcinoma cells, they also modulate the immune responses and inflammation and their inhibition may also have unwanted side effects.

To address this issue, we have concentrated in the effects of COX-2 in colon carcinoma cells. COX-2 activity regulates gene expression, which confers cells advantages for growth, migration, invasion, and metastasis (Stamatakis et al., 2015). We found that stable COX-2 overexpression in carcinoma cell lines (HT-29, HCT116, and Caco2) significantly affects gene transcription. Analysis of genes modified by COX-2 overexpression indicates that many genes involved in transcription, growth, apoptosis, angiogenesis, and migration were elevated (ArrayExpress, E-MEXP-2343). Moreover, a review of the available databases and literature in other carcinoma cell lines overexpressing COX-2 or repressed siRNA also identify some COX-2 downstream effectors responsible for the pro-tumorigenic properties of COX-2, although none of them performed a comprehensive gene expression analysis. Altogether, those analyses identified some interesting molecules. We selected some genes and validated them by quantitative RT-PCR in colon carcinoma cell lines, either induced by PGE_2_ or by overexpression of COX-2 or mPGES1. The results of mRNA levels obtained for some of those genes are shown in **Table 1**. Besides, we performed analyses of mRNA expression and survival in colon adenocarcinomas using public databases. From all these analyses some expected genes emerged, but others are relatively new and amenable to drug targeting.

**Table 1:** Genes upregulated by COX-2, mPGES1, or PGE_2_.

	Cox-2^1^	PGE_2_^2^	mPGES^3^	COAD risk^4^	Metastasis risk^5^
*PTGS2*	Increased	Increased	Increased	No	Higher
*PTGES*	Highly increased	NT	NT	Higher	Higher
*DUSP4*	Increased	Increased	NT	NO	Higher
*DUSP10*	Increased	Increased	Increased	High	Higher
*PMEPA*	Increased	Increased	NT	NO	Higher
*IL15Ra*	Increased	NT	Increased	NO	NO
*KLF4*	Increased	NT	NO	Lower	Lower
*CALD1*	Increased	NT	NT	High	Higher
*TACSTD2*	Increased	NO	NT	Weak	Lower
*NFAT5*	Increased	NT	NO	Weak	Lower
*DDIT3*	Increased	NT	NT	Higher	Lower
*Zc3h12c*	Increased	NT	NT	Lower	NO
*MMP7*	Increased	NT	NT	NO	NO
*CYR61*	Increased	NT	NT	High	Higher
*CXCL2*	Increased	NO	NO	Lower	Lower
*NEDD9*	Increased	Increased	NT	NO	NO
*PDCD4*	Decreased	Decreased	NT	NO	NO
*CXCR4*	Decreased	NT	NT	NO	Higher
*FGFR4*	Decreased	NT	NO	Lower	Lower
*NR0B2*	Decreased	NT	NT	Lower	Lower
*CA9*	Decreased	NT	NT	NO	Lower
*REG4*	Highly decreased	NT		Lower	Higher

(1) Data are obtained from RT-PCR of COX-2 overexpresssing carcinoma cells (our data on HT-29, HCT116, or Caco2 or reported in the literature) compared to control cells. (2) Data are obtained from RT-PCR of PGE2 treated carcinoma cells (our data on HT-29, HCT116 and Caco2 or reported in the literature) compared to untreated control cells. (3) Data are obtained from RT-PCR of mPGES1 overexpresssing carcinoma cells (our data on HT-29, HCT116, and Caco2) compared to control cells. (4) Associated to significant worse prognosis in databases of patients with Colon adenocarcinoma http://www.oncolnc.org/; or (5) bioprofiling database (http://www.bioprofiling.de/). NT, Not tested or reported; NO, no effect; Higher means that high expression is associated with higher risk of death or metastasis; Lower means that high expression is associated with lower risk of death or metastasis.

### Dual-Specificity MAPK Phosphatase 10

Dual-specificity Mitogen Activated Protein Kinase (MAPK) phosphatase 10 (DUSP10), also named MPK5, was found altered by COX-2 overexpression in CRC cell lines in several data sets (Doherty et al., 2009) included our own. DUSP10 is able to dephosphorylate p38 and c-Jun NH_2_-terminal kinase (JNK). Until recently, there were few reports regarding the putative role of DUSP10 in cancer. Most of those studies have found increased *DUSP10* mRNA in tumor tissue, thus suggesting a pro-tumorigenic role for this phosphatase (Jimenez-Martinez et al., 2019b). In CRC, the expression of certain DUSP10 polymorphisms are linked to an enhanced CRC risk (Duan et al., 2015).

We found that DUSP10 overexpression increased the growth of CRC cell lines and mouse xenografts, while the opposite phenotype was observed by DUSP10 silencing. Cancer progression is related to an uncontrolled cell division and DUSP10 overexpression produces the loss of cell-contact inhibition through the dephosphorylation of Yes-associated protein (YAP) at Ser397. This dephosphorylation retains YAP in the nucleus. In fact, we found that high amounts of DUSP10 and YAP1 are located in the nucleus of CRC cells (Jimenez-Martinez et al., 2019a). Additionally, the quantity of nuclear DUSP10 in CRC tumor biopsies is directly correlated with high tumor stage CRC and poor prognosis and survival in a large cohort of CRC patients, being also associated to high expression of nuclear YAP1. All these data point at DUSP10 as a downstream protein in COX-2 signaling and provide evidence of the role of DUSP10 in CRC progression *via* YAP1 regulation (Jimenez-Martinez et al., 2019a). Interestingly, an inhibitor of DUSP10 have been described (Hommo et al., 2015), supporting its use in CRC.

### Dual-Specificity MAPK Phosphatase 4

Another dual-specificity MAPK phosphatase, DUSP4/MKP-2, was also upregulated by COX-2. DUSP4 role in several cancers has been documented (Low and Zhang, 2016; Seternes et al., 2019). An altered expression of DUSP4 is related to colon tumorigenesis. Thus, elevated DUSP4 expression is associated with microsatellite instability in CRC patients (Gröschl et al., 2013) being also associated with liver and lung metastases of CRC (Saigusa et al., 2013). Moreover, DUSP4 overexpression in CRC cell lines produced upregulation of MAPK targets, such as EGR1, FOS, and MYC, and downregulation of the mismatch repair gene MSH2, all these effects leading to an increase in cell proliferation (Gröschl et al., 2013). In contrast, it has been more recently reported that the decrease in the expression of DUSP4 in CRC cell lines activated ERKs, causing cell proliferation and invasiveness (Ichimanda et al., 2018).

Interestingly, DUSP4 overexpression in CRC cell lines decreases their sensitivity to doxorubicin, a drug used to treat CRC (Kang et al., 2017). Moreover DUSP4 genetic inactivation increased the resistance to cetuximab, an epidermal growth factor receptor (EGFR)-blocking antibody, widely used and approved for treatment of metastatic CRC in cell lines and to another well‐known EGFR inhibitor, erlotinib (Park et al., 2019). Interestingly, those effects are also related to COX-2 expression, since *DUSP4* was the highest induced gene in cetuximab-resistant CRC cells (Lu et al., 2016). All these data together point at DUSP4, enzyme regulated by COX-2, as a factor whose overexpression leads to CRC development and invasion, and which can be a promising therapeutic target. Moreover, DUSP4 specific inhibitors have been described (Park et al., 2014) supporting its clinical testing in CRC at least in some drug resistant tumors.

### Trophoblast Cell-Surface Antigen 2

Trophoblast cell-surface antigen 2 (TROP2), also called Tumor-Associated Calcium Signal Transducer 2 (TACSTD2), is a cell-surface glycoprotein expressed during embryonic and fetal development which is involved in cell proliferation, cell binding, motility, and metastasis (Mcdougall et al., 2015). TROP2 overexpression can induce cancer growth and is associated with poor prognosis and drug resistance in cancer cells (Shvartsur and Bonavida, 2015).

Our COX-2, but not mPGES1, overexpressing colon carcinoma cells present high levels of *TACSTD2*. Besides, high expression of *PTGS2* and *TACSTD2* genes have been found also associated in lung cancer metastasis (Citterio et al., 2012). Previous studies in CRC patients, already showed the correlation between high expression of TROP2 and metastasis in CRC (Kapoor, 2013). The overexpression of TROP2 together with MMP7, another COX-2 induced gene (see below), is a predictor of worse prognosis and relapse in CRC (Fang et al., 2009). TROP2 expression enhances anchorage-independent growth in colon carcinoma cell lines (Wang et al., 2008) and its activation of TROP2 by Tumor Necrosis Factor (TNF)-alpha induces cell migration and invasion (Zhao and Zhang, 2018).

More importantly, an anti-TROP-2 antibody, sacituzumab govitecan, is a potential therapeutic drug for metastatic solid tumors (Starodub et al., 2015). Since TROP2 is consistently induced by COX-2 overexpression, this makes it an alternative target to COX-2 blockade therapy in CRC.

### Matrix Metalloproteinase- 7

Matrix metalloproteinases (MMPs) are proteolytic enzymes which degrade and remodel the extracellular matrix (ECM) in physiological processes, such as in cell migration, and have also been involved in metastasis (Zucker and Vacirca, 2004). It has been reported that MMP7 is one of the most important MMPs in colorectal tumorigenesis, promoting angiogenesis, invasiveness, and tumor survival. Thus, MMP-7 may enhance CRC progression due to its proteolytic activity on several cell surface molecules as EGFR, Fas-L, etc. (Wang et al., 2006). MMP7 is abundantly synthesised by colon carcinoma cells (Zucker and Vacirca, 2004). A study of CRC patients demonstrated an increase in COX-2 and MMP-7 expression when compared to normal tissue and colon polyps sample (Bengi et al., 2015). High levels of MMP-7 together with PTEN down-regulation where detected in CRC and were related to tumor stage and progression (Bi et al., 2013). Moreover, COX-2 overexpression in carcinoma cells modulates the adhesive properties of MMPs (Tsujii and Dubois, 1995; Tsujii et al., 1997). PGE_2_ can transactivate EGFR thereby inducing the proliferation of CRC cell lines and exerts its functions in part through molecules such as MMP-7 (Pai et al., 2002; Karpisheh et al., 2019).

### Krüppel-Like Factor 4 (KLF4)

Krüppel-like factor 4 (KLF4) is a transcription factor that was firstly identified as a regulator of cell growth arrest being one of the most important factors in Cancer Stem Cells (CSC). It is expressed in differentiating cells and it is also known to have a role in apoptosis suppression (Ghaleb and Yang, 2017). KLF4 mRNA is consistently and strongly induced in our COX-2 but not in mPGES1 overexpressing colon carcinoma cells and it was shown that COX-2 and KLF4 colocalized in carcinoma cells of CRC tumor samples (Shao et al., 2008). Moreover, in HT-29 colon cancer cells, the expression of KLF4 was induced by 15-Deoxy-Delta-12, 14 PGJ_2_, a downstream product of COX-2 signaling pathway (Chen and Tseng, 2005).

It has been described that the levels of high KLF4 mRNA in normal tissues can be used as a prognostic indicator of survival in CRC patients (Lee et al., 2014), suggesting a regulatory role in CRC progression. In contrast, other reports have indicated a protumoral role of KLF4 in CRC. Thus, KLF4 was found in colon adenocarcinoma metastasis to the liver (Humphries et al., 2018) and low KLF4 expression was found in poorly differentiated CRC tissues (Hu et al., 2011). A study performed in cancer stem cell (CSC)-enriched spheroid CRC cell lines revealed a role of KLF4 in the invasiveness, migration, resistance to treatment, and ability to generate tumors as well as in the induction CSCs markers in those cells (Leng et al., 2013).

### Programmed Cell Death 4 (PDCD4)

The expression of programmed cell death 4 (PDCD4), a tumor suppressor protein that inhibits tumor development in many cancer types, is decreased in CRC and this downregulation in colon tumors has been related to worse prognosis (Long et al., 2019). Several studies correlate PDCD4 overexpression with inhibition of cell proliferation and invasion in different tumor cancer cell types. In colon carcinoma cells, high levels of PDCD4 inhibited Akt signaling pathway, decreasing invasiveness (Wang et al., 2017). Moreover, a COX-2 inhibitor, NS-398, increases PDCD4 in HCA-7 cells, a colon cancer cell line (Zhang and Dubois, 2001), in agreement with our observation that is consistently downregulated by COX-2 overexpression in carcinoma cell lines. The overexpression of COX-2 in response to inflammation induces an increase in PGE_2_ production and PGE_2_ decreases PDCD4 protein levels through upregulation of miR-21 (Peacock et al., 2014). MiR-21 role in cancer has been extensively studied and its upregulation may explain the intrinsic resistance of some cancers to chemotherapy (Pfeffer et al., 2015), being miR-21 and PDCD4 inversely correlated in CRC (Chang et al., 2011; Horiuchi et al., 2012). Thus increasing PDCD4 levels through pharmacological manipulation of miR-21 could represent a novel therapeutic strategy in the treatment of CRC, as an alternative to COX-2/PGE_2_ blockade in CRC.

### Nuclear Factor of Activated T-Cells 5

Nuclear Factor of Activated T cells- 5 (NFAT5) is a transcription factor belonging to the NFAT family whose expression is upregulated by osmotic stress to difference with the other four members of this family (Qin et al., 2014). Dysregulation of NFAT signaling has been linked to tumor progression in several cancers (Qin et al., 2014). Interestingly, NFAT is a key transcription factor regulating COX-2 expression in CRC cell lines (Duque et al., 2005), suggesting the existence of a positive feedback loop between NFATs and COX-2. However, little is know on NFAT5 and CRC. We found NFAT5 is induced by COX-2 overexpression and it has been described that promotes metastasis through the induction of alpha(6)beta(4) integrin in human breast and colon tumors (Jauliac et al., 2002), and besides some associations between NAFT5 SNPs and CRC risk has been found (Slattery et al., 2011). NFAT5 expression has also been associated to metastasis in other cancer types and to proliferation of lung adenocarcinoma cells (Guo and Jin, 2015).

## COX-2 and the TGF-β Pathway

Among the genes induced by COX2, we found in our arrays many genes of the TGF-β pathway, which we later confirmed by PCR (**Table 2**). Many of them are also induced by PGE_2_ treatment although in a much lower extent, suggesting that other COX2 derived products, as PGF_2α_, are also involved. Apart from those, some other genes induced by TGF-β were also induced by COX-2/PGs.

**Table 2:** Induction of TGFβ pathway or p53 pathway genes by COX-2 overexpression or PGE_2_ treatment in HT-29 colon carcinoma cells.

TGFβ pathway	COX-2	PGE_2_
*TGFB1*	+ 24 ± 4	+ 3 ± 1
*TGFBR1*	+ 18 ± 3	+ 3 ± 0.8
*TGFBR2*	+ 14 ± 3	+ 2 ± 0.4
*SMAD1*	+ 20 ± 8	+ 2 ± 0.6
*SMAD3*	+ 38 ± 3	+ 3 ± 0.5
*SMAD5*	+ 35 ± 6	+ 3 ± 0.5
*SERPINE1*	+ 7 ± 2	
*EDN1*	+ 25 ± 4	+ 2 ± 0.2
*ID1*		+ 2 ± 0.3
*ENG*	+ 36 ± 9	
*CTGF*	+ 2 ± 0.3	+ 3 ± 0.5
**p53 pathway**		
*TP53*	+ 10 ± 2	+ 4 ± 1
*MDM2*	+ 28 ± 3	+ 3 ± 1
*CDKN1A*	+ 7 ± 1	
*TGFA*	+ 46 ± 8	
*GADD45*	+ 20 ± 3	+ 2 ± 0.5
*PCNA*	+ 8 ± 1	+ 2 ± 0.3
*BAX*	+ 23 ± 4	+ 2 ± 0.8
*FASL*	+ 25 ± 2	
*TSP1*	+ 34 ± 2	+ 3 ± 0.7

Fold induction of indicated mRNA in HT-29 stably overexpressing COX-2 respect empty vector transfected cells or HT-29 treated with 1 µM PGE_2_ for 24 h. Results shown are the mean ± SD of 3 independent experiments.

One of them was Prostate Transmembrane Protein, Androgen Induced 1 (PMEPA1, also known as TMEPA1, transmembrane prostate androgen induced 1). PMEPA1 was first identified as being upregulated in renal cell carcinoma and was designated as solid tumor associated gene 1 (STAG1) (Rae et al., 2001). PMEPA1 is known to be induced by TGF-β and modulates TGF-β signaling by competing with SARA (SMAD Anchor for Receptor Activation) for R-SMAD binding to sequester R-SMAD phosphorylation and promoting lysosomal degradation of TGF-β receptor (Watanabe et al., 2010). PMEPA1, through a negative feedback loop, is described as the responsible to convert TGF-β from a tumor suppressor to a tumor promoter in breast cancer (Singha et al., 2010).

We have demonstrated that PMEPA1 is a COX-2, PGE_2_, PGF2α, and calcium (through Ionophore treatment) -induced gene in colon and ovary cancer cells (Jimenez-Segovia et al., 2019). Furthermore, PMEPA1 is involved in differentiation of colon epithelial cells and high levels are also found in all phases of CRC development, including metastasis, in patients (Brunschwig et al., 2003). Furthermore, its high expression in CRC is related to poor prognosis and postulated as a predictor of relapse risk (Xu et al., 2017; Zhang et al., 2019). PMEPA1 is also overexpressed in intestinal tumors in Apc (Min) mice, which are prone to intestinal adenoma formation and is also dysregulated in human CRC adenomas (Reichling et al., 2005).

A recent study using colon cancer cell lines revealed the role of PMEPA1 in cell migration and invasion through bone morphogenetic proteins (BMP) signaling pathway activation and phosphorylation of the transcription factors Smad1 and Smad5 (Zhang et al., 2019), which also correlates with its described effect in ovarian cancer cells (Jimenez-Segovia et al., 2019).

NEDD9 (Neural Precursor Cell Expressed, Developmentally Down-Regulated-9) or HEF1 (Human enhancer of filamentation 1) is a structural protein mainly found in epithelial cells, which is upregulated by TGF-β and expressed transiently during embryonic life in mice. Many functions have been attributed to NEDD9, among them, the transmission of growth control signals between focal adhesions. It has also been involved in melanoma tumorigenesis and, most notably, in metastasis (O’neill et al., 2007; Tikhmyanova et al., 2010; Lee et al., 2011).

NEDD9 was also identified as one of key genes in CRC, being upregulated in CRC tumors (Li et al., 2011; Cui et al., 2017). Interestingly, two independent groups have shown that NEDD9 overexpression elicited the same effects as PGE_2_ treatment on cell proliferation, cell cycle progression, colony formation, migration, and xenograft tumor growth in colon carcinoma cell lines (Xia et al., 2010; Li et al., 2011). Interestingly, those PGE_2_ effects were reversed by inhibition of NEDD9 expression, indicating that NEDD9/HEF-1 could be an important downstream mediator in the pro-tumoral activity of PGE_2_ in CRC. In this sense, it would be possible to develop inhibitors to disrupt NEDD9 interactions with oncogenic partners (Tikhmyanova et al., 2010).

Cysteine Rich Angiogenic Inducer 61 (Cyr61/CNN1) is another TGF-β regulated gene (Han et al., 2016). It is a member of the CNN family of extracellular matrix associated proteins, which induces cell division and differentiation, chemotaxis, angiogenesis, and adhesion. In CRC, high levels of this protein induce migration and increase invasiveness (Chang et al., 2014). Cyr61/CNN1 has been described as potential serum marker for CRC (Song et al., 2017), whereas Cyr61 expression in the tumor tissue indicates poor prognosis in colon cancer patients being statistically associated with greater mortality (Jeong et al., 2014). Altered activation of CYR61 gene enhancers occurs during CRC development, being CYR61 expression induced by FOXA1 and CBP in colon cancer cells pointing at both transcription factors as targets for CRC treatment (Xie et al., 2019). Interestingly, PGF2α through FP receptors can upregulate the expression of CYR61 mRNA (Xu et al., 2009). We also found a significant positive correlation of COX2, DUSP10 with CYR61 in colon carcinoma cell lines (Jimenez-Martinez et al., 2019a).

Caldesmon (CaD), a major actin-associated protein, is found in smooth muscle and non-muscle cells with contractile function. In normal cells, CaD regulates contraction through the binding to two of the components of the thin filaments, actin, and tropomyosin, in the presence of Ca^2+^ (Wang and Coluccio, 2010). CaD expression was reported in both adenomas and adenocarcinomas (Porter et al., 1993). There are two alternatively spliced isoforms of CaD, high (h-CaD) and low (l-CaD), which differ in their molecular weight. H-CaD levels are lower in the late stages of colorectal adenocarcinoma while the low-molecular weight variant expression varies during tumor development and it is related to metastasis (Köhler, 2011; Kim et al., 2012). CaD is absent in the stroma in CRC but present in smooth muscle, thus it is a marker for desmoplasia and tumor invasiveness of the large intestine’s muscularis propria (Roberts et al., 2014). It has been also described that CaD mRNA levels are higher in colon carcinoma cell lines than in non-transformed normal colon epithelial cells (Nimmrich et al., 2000).

However, little is known about the role of the protein in the carcinogenic process. CaD is an important regulator of podosome formation which is involved in the degradation of the extracellular matrix (ECM), promoting in this way cell invasion, that is required for metastasis (Yoshio et al., 2007). L-CaD overexpression promotes expression of the regulatory p21 and c-PARP and suppresses NF-κB and phosphorylated mTOR expression, whereas its suppression increases sensitivity to chemo- and radiotherapy in colon carcinoma lines (Kim et al., 2012). TGF-β1 regulates the expression and phosphorylation of CaD in epithelial cells modulating epithelial-mesenchymal (EMT) transition (Nalluri et al., 2018).

## COX-2 and the p53 Pathway

The role of p53 in cancer in general and CRC in particular is well known [reviewed in (De Moraes et al., 2007; Watson and Collins, 2011)]. Mutations in the tumor suppressor gene TP53, which encodes the protein p53, are frequently found in human cancers. Mutations in K-ras, adenomatous polyposis coli (APC), and p53 induce the transition from healthy colonic epithelia to CRC (Dixon et al., 2013).

Among the genes induced by COX-2, we found in our arrays many genes of the p53 pathway, as it was found for the TGF pathway, that were also later confirmed by PCR (**Table 2**). Among those are TGFα, as well as others involved in cell cycle, apoptosis, DNA repair, and angiogenesis that my explain some of the diverse pro-tumoral activities of COX-2 in CRC. Again some of them were also induced by exogenous addition of PGE_2_ but in lesser extent, further suggesting that other prostaglandins, likely PGF_2α_, may be involved.

In addition, there is a well-known crosstalk between p53 and COX-2, in which COX-2 decreases p53 transcription, and p53 also regulates COX-2 expression (De Moraes et al., 2007). Furthermore, many COX-2 inducers, such as hypoxia, cytokines, oncogene activation, carcinogens, and inflammation, can also activate p53. The role of p53 in the regulation of COX-2 expression and activity has been extensively described. Thus, it has been reported the increase in COX-2 transcript in human tumor cells expressing p53 (Han et al., 2002; De Moraes et al., 2007). COX-2 upregulation by p53 has been attributed to an increase on the binding of NF-κB to COX-2 promoter in response to p53 overexpression (Benoit et al., 2006). Conversely, COX-2 avoids the transcriptional activity of p53 on target genes (De Moraes et al., 2007). COX-2 is not only regulating p53 function but also its subcellular localization, as it was demonstrated in human colon cell lines treated with celecoxib, a COX-2 inhibitor, in which p53 nuclear location was promoted (Swamy et al., 2003). Interestingly, joint nuclear expression of COX-2 and p53 was significantly associated with adenoma recurrence in CRC (Brand et al., 2013).

Nonetheless there is less reported evidence of COX-2 effects on the p53 pathway. Interestingly our analyses have defined many p53 related genes that are modulated by COX-2 overexpression in colon carcinoma cells. Many of them were not described before as modulated by COX-2 and can provide clues in new therapeutic approaches.

## Conclusion

**Figure 2:**
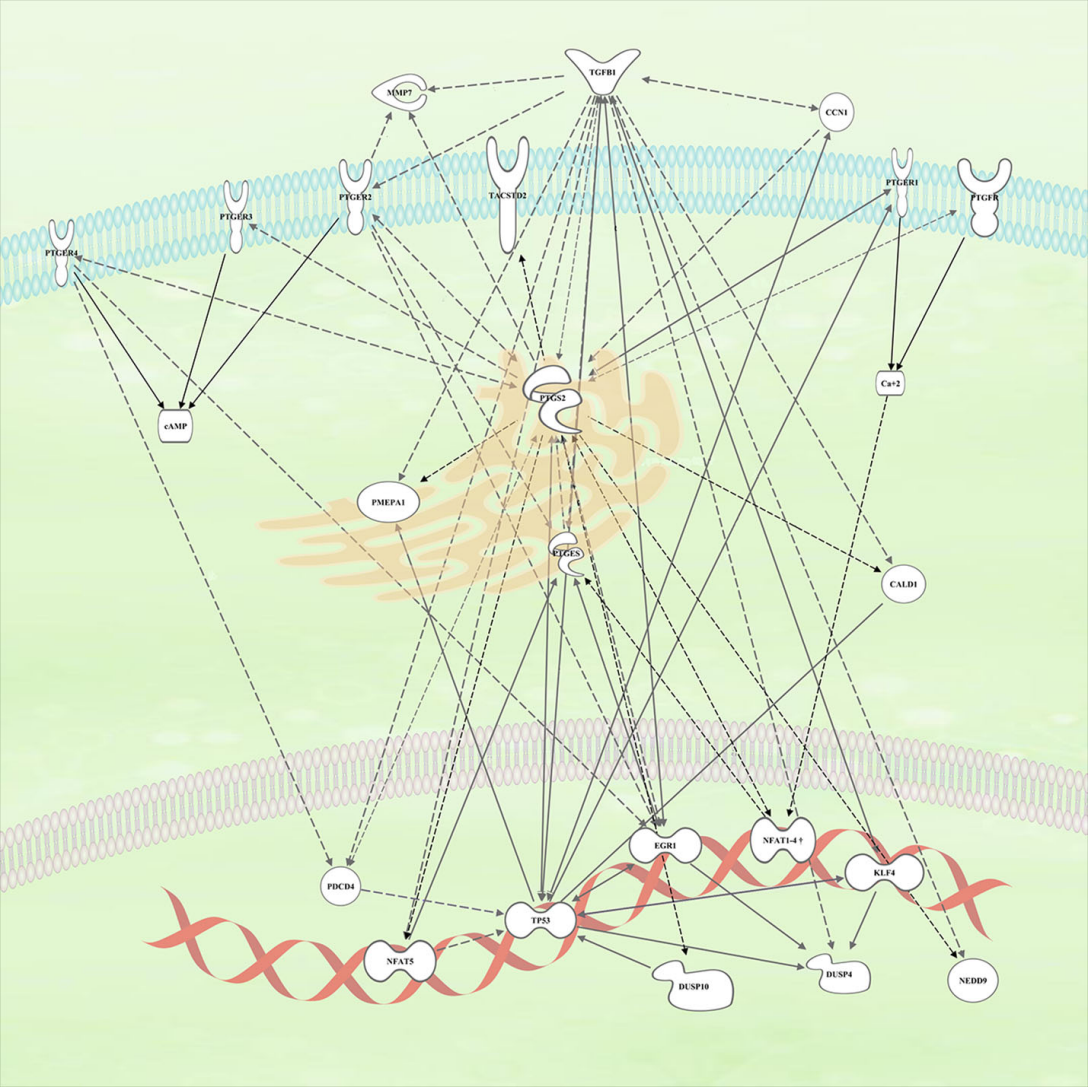
Connection within the different genes upregulated by COX2 and likely involved in colorectal cancer (CRC) tumors. The continuous arrows are of direct interaction/activation while the discontinuous ones are indirect activation in which intermediaries participate.

COX-2 has been identified through clinical trials, epidemiological meta-analyses, and preclinical models and as a key molecule in the development of CRC. However, the use of specific COX-2 inhibitors in the clinic has been hampered by their side effects. The identification of genes regulated by COX-2 activity has allowed us to identify some genes that have been already implicated in several aspects of CRC development and therapy. The relationships among those genes and with the PG pathways are summarized in **Figure 2**.

Of all molecules described here, some of them are more amenable to drug discovery. Besides some molecules had already specific inhibitors developed, as the phosphatases DUSP10 and DUSP4, as well as monoclonal antibodies to TROP2. This, together with the clinical evidence available in CRC, pointed out to these 3 molecules as the most suitable to be considered as putative drug targets and to engage in clinical trials especially in drug resistant settings or advanced CRC stages most likely in combination therapy. Also the interaction of the TGF-β and COX2 pathway deserve further clinical exploration. In summary, drugs targeting the COX-2 downstream molecules described will likely lack the unwanted side effects of COX-2 pharmacological inhibitors, providing alternative approaches in colon cancer.

## Author Contributions

Writing—review and editing: MF, AH-E, KS, MJ-M, and RL-P. Review of published literature: MF, AH-E, KS, MJ-M, and RL-P. Analysis of the data in figures and tables: KS.

## Funding

This research was funded by grants from “Ministerio de Ciencia e Innovación” (SAF2013-42850-R and SAF2016-75988-R) “Comunidad de Madrid (S2017/BMD-3671. INFLAMUNE-CM), Fondo de Investigaciones Sanitarias” (BIOIMID) to MF and Institutional grants from “Fundación Ramón Areces” and “Banco de Santander”. KS was the recipient of a Spanish Association Against Cancer Oncology Investigator grant (AECC AIO).

## Conflict of Interest

The authors declare that the research was conducted in the absence of any commercial or financial relationships that could be construed as a potential conflict of interest.
